# Physical Stimulation Combined with Biomaterials Promotes Peripheral Nerve Injury Repair

**DOI:** 10.3390/bioengineering9070292

**Published:** 2022-06-30

**Authors:** Zhipeng Zeng, Yajing Yang, Junyong Deng, Muhammad Saif Ur Rahman, Chengmei Sun, Shanshan Xu

**Affiliations:** 1 Institute for Advanced Study, Shenzhen University, Shenzhen 518060, China; 2021110013@bsu.edu.cn (Z.Z.); fiasanar@szu.edu.cn (M.S.U.R.); 2 Key Laboratory of Optoelectronic Devices and Systems of Ministry of Education and Guangdong Province, College of Physics and Optoelectronic Engineering, Shenzhen University, Shenzhen 518060, China; 3 School of Sport Medicine and Rehabilitation, Beijing Sport University, Beijing 100084, China; 4 National Cancer Center, National Clinical Research Center for Cancer, Cancer Hospital and Shenzhen Hospital, Chinese Academy of Medical Sciences, Peking Union Medical College, Shenzhen 518116, China; zhouxuchang@bsu.edu.cn; 5 Department of Rehabilitation, Second Affiliated Hospital of Nanchang University, Nanchang 330006, China; ndefy19446@ncu.edu.cn

**Keywords:** peripheral nerve injury, biomaterial, physical stimulation, electrical stimulation

## Abstract

Peripheral nerve injury (PNI) is a clinical problem with high morbidity that can cause severe damage. Surgical suturing or implants are usually required due to the slow speed and numerous factors affecting repair after PNI. An autologous nerve graft is the gold standard for PNI repair among implants. However, there is a potential problem of the functional loss of the donor site. Therefore, tissue-engineered nerve biomaterials are often used to bridge the gap between nerve defects, but the therapeutic effect is insufficient. In order to enhance the repair effect of nerve biomaterials for PNI, researchers are seeking to combine various stimulation elements, such as the addition of biological factors such as nerve growth factors or physical factors such as internal microstructural modifications of catheters and their combined application with physical stimulation therapy. Physical stimulation therapy is safer, is more convenient, and has more practical features than other additive factors. Its feasibility and convenience, when combined with nerve biomaterials, provide broader application prospects for PNI repair, and has therefore become a research hot spot. This paper will review the combined application of physical stimulation and biomaterials in PNI repair in recent years to provide new therapeutic ideas for the future use of physical stimulation in PNI repair.

## 1. Introduction

Peripheral nerve injury (PNI) is a clinical problem that leads to the loss of peripheral nerve structure and/or function and partial or complete loss of sensory, motor, and autonomic nerve functions due to accidents, trauma, surgery, and other reasons [1]. It is reported that there are about a million cases of peripheral nerve injury in the world every year [2], which causes a heavy medical burden for society [3]. Although PNIs can achieve nerve regeneration, there are many limitations in the nerve regeneration process, so surgery is generally required after PNI [4,5]. Autologous nerve grafts are considered the gold standard in PNI treatment [6,7]. However, there are still many problems, such as insufficient sources, sequelae after removal of the donor, artificially formed traumatic neuromas, etc. [8]. Due to the above limitations, actively seeking alternative treatment strategies for autologous nerve transplantation has become a research hotspot in nerve repair. In recent years, numerous studies showed that the development of artificial nerve grafts is considered a promising alternative to autologous nerve grafts [9,10,11,12,13].

As a promising treatment method, a tissue-engineered nerve graft can bridge the peripheral nerve gap and promote nerve regeneration [14,15]. A series of nerve grafts based on biomaterials have been developed in the laboratory and clinic, mainly nerve guidance conduits (NGCs) based on natural polymers and synthetic polymers [16,17,18]. However, the therapeutic effect of PNI repair is unsatisfactory. To improve the treatment effect, several different stimulating factors have been incorporated into NGCs, including biological components (such as Schwann cells, S.C.s) [19] or neurotrophic factors [20], physical stimulators (such as conductivity [21]), surface modifications (such as terrain guides), and physical simulation therapy [22]. Although the addition of different factors such as biological components and surface modification play a specific role in promoting PNI repair, there are many problems such as immune rejection, transportation and preservation of biological components, ethical problems, and high manufacturing costs [23], promoting the in-depth study of the combination of safe and convenient physical stimulation alongside nerve biomaterial grafting.

Physical stimulation can provide a non-pharmacological means of treatment to restore the function of damaged tissues and organs. Considering the importance of nerve axons in neuroscience treatment, many actively controlled physical stimulation methods, such as electrical stimulation [24], light stimulation [25], mechanical energy [26], and magnetic stimulation [27], have been applied to promote the growth of nerve axons. Given this, considering the difference in the effect of promoting neuronal growth and improving the recovery of PNI function, the difference in the mechanism of action and the difference in the possibility and feasibility of future applications after various physical stimulation therapies are combined with the use of biomaterials. By reviewing the related research articles on PNI repair via physical stimulation combined with biomaterials in recent years, this paper is expected to provide ideas for further exploring their combined effects.

## 2. Application of Electrical Stimulation in Biomaterials for Peripheral Nerve Injury Repair

In 1983, Nix and Hopf first investigated the role of electrical stimulation (E.S.) in promoting nerve regeneration [28]. Preclinical studies in subsequent decades and recent prospective randomized clinical trials further proved that E.S. can enhance nerve axon regeneration and accelerate functional recovery [29,30,31]. There are two main methods of E.S. for PNI treatment, transcutaneous electrical nerve stimulation and electrode implantation devices. The former has a limited stimulation effect on deep muscles [32]; the latter requires a second operation, bringing additional costs and complications [33]. In addition, cumbersome external electrical control devices are required, which reduce the convenience of use; penetrating electro-acupuncture and/or built-in conductive NGCs are required by electrode implantation devices, which often cause various complications (such as inflammation, immune rejection, pain, etc.) and reduce the effectiveness due to mechanical mismatch and charge overflow problems [25,26]. Researchers provide new ideas and solutions for solving the above problems by developing new nerve E.S. biomaterial devices, such as self-powered nerve scaffolds and wireless nerve stimulators.

### 2.1. Self-Powered Nerve Scaffold

**Figure 1:**
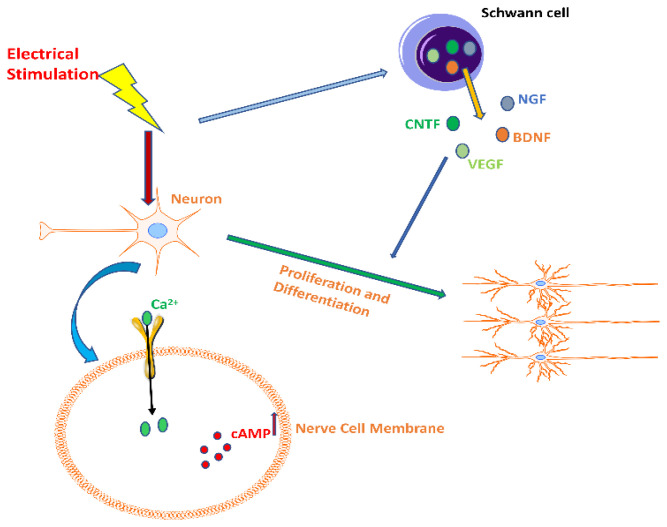
The effect of electrical stimulation on neurons. Electrical stimulation increased the secretion levels of nerve growth factor (NGF), brain-derived neurotrophic factor (BDNF), ciliary neurotrophic factor (CNTF), and vascular endothelial growth factor (VEGF) in Schwann cells, enhancing the activity of Ca^2+^ channels in nerve cell membranes, which in turn activated related effector proteins and increased cAMP levels, ultimately promoting neuronal proliferation and differentiation.

Self-powered means that the device can generate electric energy without using an external power supply during operation [34]. As the advantages of bio-conductive scaffolds, such as cost-effectiveness and reproducibility, have gradually emerged, some scholars have proposed integrating self-powered functions and scaffolds to design a self-powered nerve scaffold [35], which can better combine the advantages of both [36]. Mohseni et al., designed a novel self-electrically stimulating porous scaffold based on natural polymers [10], but they did not further validate its therapeutic effects in vivo and elucidate its mechanism. Another study bridged the gap. This study reports a nerve-guiding catheter containing biocompatible MgFeMn primary cells [35]. The scaffold showed an excellent therapeutic effect that can be attributed to the direct current provided by the primary cell, stimulating the regeneration of S.C.s, upregulating the levels of various growth factors, enhancing the activity of Ca^2+^ channels, and activating the relevant effector proteins, thus promoting neuronal growth (as shown in Figure 1) [37,38]. The scaffold is exceptionally designed with a 2–3 day battery life due to the lack of raw materials. The use of glucose and oxygen in the human body to generate electricity has a long history [39] and is expected to solve the problem. Sun et al., used this principle to prepare a self-powered nerve scaffold and applied it to an animal model with good results, but observed a slight foreign body reaction [32]. This study takes advantage of the abundant glucose and oxygen content in the body and provides a new direction for the source of self-powered materials.

Self-powered nerve scaffolds are a promising direction for PNI repair, and the maturity of this technology could allow for great convenience for clinical applications. Relevant studies have begun to use natural polymers, metal materials, or body fluid components to create these kinds of scaffolds. However, there are still some problems to be solved. The long-term use of metal materials in the body may have toxicity risks for cells or tissues or cause rejection reactions, and there is also the lifespan problem caused by rapid consumption of metal. Therefore, choosing non-toxic metal materials for power supply materials or developing metal electrodes that can generate electricity from the substances of the body (e.g., oxygen and glucose) may solve the problem of a long-term, safe, and adequate power supply for metal materials.

### 2.2. Wireless Nerve Stimulator

Although the effect of E.S. on promoting PNI repair is clear, how to transmit electrons more effectively at the site of nerve injury, how to control the treatment time window accurately, and how to avoid infection effectively have always been important issues in the application of E.S. in PNI. A wireless nerve stimulator offers a lead-free, wirelessly controllable solution with a reduced risk of chronic infection [40]. MacEwan et al., created the first implantable wireless nerve stimulator that works similarly to wired electrodes and successfully delivers electrical signals to injured nerve tissue, accelerating nerve function repair [40]. It was also found that wireless nerve stimulators promote the differentiation of neural stem cells (NSCs) into neurons and the formation of nerve axons [41]. In addition, an inductively powered nerve stimulator placed on cuff electrodes was developed, which was able to activate peripheral nerves to induce muscle fiber recruitment [42]. This device exhibits good power supply performance. However, the necessity of regulating the current means that it is not a convenient method. A study designed a self-powered wireless neurostimulator that could be controlled by external programming, with better electrical signal regulation and a deeper depth of action [43]. None of the above studies considered the tissue inflammatory response caused by the mechanical mismatch due to electrode hardness, which can lead to the encapsulation of the implanted electrode by scar tissue and eventually cause the device to fail [44]. Therefore, it is still crucial to develop materials that can deform with nerves, muscles, and other soft tissues. Cuttaz et al., developed a nerve cuff electrode with conductive polymer (C.P.) composites. After testing, it was found that the device has good stability, stretchability, and cytocompatibility [45]. To verify the therapeutic effect of flexible electrodes further, some scholars prepared an implantable, wirelessly controlled flexible microelectrode array (MEA) [46]. The study found that the device had good mechanical compliance, successfully prevented muscle atrophy and degeneration of acetylcholine receptors after PNI in rodents, and recorded electrical activity in the epimysium. The study validates a new concept of the flexible electrode that integrates with therapeutic and diagnostic capabilities, but the electrode is non-absorbable and requires a second surgery to remove. To solve this problem, scholars further developed absorbable nerve electrodes. Guo et al. fabricated a bilayer biocompatible, wireless bioabsorbable nerve stimulator [47]. The study found that the device could operate stably in mice for 5 h, meeting the 1-h E.S. criterion to promote nerve repair [48,49] and was bioabsorbable within 10 days. This device can supplement the therapeutic effect of surgery; however, the treatment time is short and may not achieve the desired effect of PNI repair. To overcome this limitation, a new wireless, peripheral electrical nerve stimulator with a treatment time beyond the intraoperative window of 6 days was developed [50]. The study found that the therapeutic effect of the device was comparable to that of a traditional electrical stimulation scaffold. The design of this study lays the engineering foundation for a broader class of bioabsorbable electronic implants, but for nerve defects with long gaps (≥3 cm), 6 days of treatment may not be sufficient to regenerate the length of the nerve across this gap. Choi et al., prepared stretchable and wireless bioabsorbable cuff electrodes to address this issue. The study found that the electrode has good electrical reliability and mechanical stability. It ran reliably for more than 30 days and significantly reduced the effects of muscle atrophy and loss of function caused by denervation [33].

In summary, wireless nerve stimulators can be wirelessly controlled to modulate the current output to achieve a more desirable therapeutic effect, which is of great significance for the treatment of PNI repair in the clinic. With the development of electronic manufacturing technology, multifunctional wireless nerve stimulators containing features such as miniaturized, flexible, and bioabsorbable electrodes have become a reality, and some even contain dual roles of therapy and EMG detection. In addition, it remains important to extend the lifetime of electrical stimulators further more effectively. Therefore, further experiments are needed to optimize various performances of wireless nerve stimulators, such as long lifespan, etc.

## 3. Application of Mechanical Energy in Biomaterials for Peripheral Nerve Injury Repair

**Figure 2:**
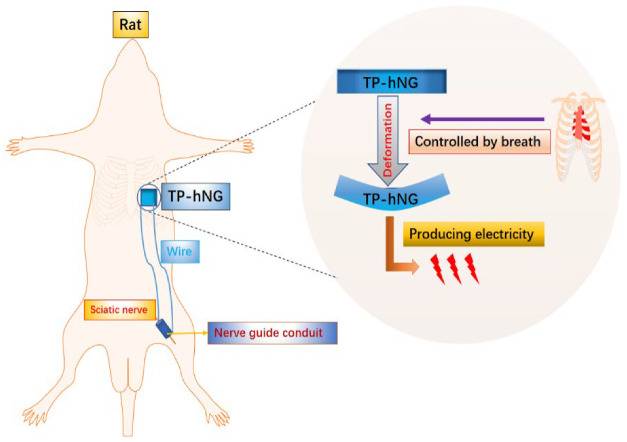
Physiologically self-regulated, fully implantable, battery-free system for PNI repair.

A nanogenerator (N.G.) is a device that converts biomechanical energy generated by mechanical stimulation such as pressure and friction into electrical energy [51]. According to its power generation principle, N.G.s can be divided into piezoelectric nanogenerators (PENGs) and triboelectric nanogenerators (TENGs) [52]. N.G.s have self-powered properties, and a scaffold with electrical conductivity is essential for recovery in PNI [53]. Therefore, the development of a N.G.-based self-powered nerve scaffold is a promising avenue. Guo et al., combined TENG and a scaffold to construct a step-driven self-powered electrical stimulation system and found that the system can enhance the proliferation and differentiation of mesenchymal stem cells (MSCs) [54]. The protocol of this study is one of the effective ways to achieve efficient tissue regeneration, but polythiophene used in the study can cause severe biological toxicity in vivo [55]. A safe long-term implantable ZnO/PCL piezoelectric nanogenerator scaffold was fabricated to avoid a malignant outcome. The device produces E.S., driven by rat movement, which in turn has the additive effect of motor promotion, E.S., and scaffold properties on peripheral nerve recovery [56]. The E.S. generated in this study has the potential for widespread application in humans; however, the magnitude of the current generated under the motor drive is prone to instability and such stimulation may adversely affect PNI repair because neurons are vulnerable to injury due to inertia at constant charge density and excessive charge injection [57]. To address this problem, Jin et al., constructed an implantable, respiratory-driven, physiologically self-regulated electrical signal (PSR-ES) nerve electrical stimulation system based on the regulation of respiratory movements by the autonomic nervous system (ANS), which was applied to a rat sciatic nerve deficit model (as shown in Figure 2) [58]. It was found that the system can promote the recruitment of chemokine receptors (such as CXCR4 and CXCR2) and that the increase in related cells and protein levels leads to the formation of micro-vessels and the regeneration of nerve fibers, finally achieving a nerve recovery effect similar to that of autologous nerve transplantation. This device rationally utilizes biomechanical energy and has ideal therapeutic effects, which will help promote the wide application of artificial biomedical electronics in regenerative medicine and drug delivery.

A N.G. scaffold combines the self-powering characteristics of a N.G. and the characteristics of nerve scaffold, which can effectively improve the effect of PNI repair, and may even represent a new gold standard, which will significantly encourage future clinical applications. The role of exercise in promoting PNI repair has been clarified, and exercise is indispensable in daily human life. On the other hand, breathing occurs 10,000 times a day. If the body’s exercise can be reasonably combined with biological materials, it can save energy and scientifically adjust the rhythm of electrical stimulation or improve the therapeutic effect. This combined treatment mode has brought about new ideas for the treatment of PNI and can even bring hope to the broader promotion of biomedical technology in the future.

## 4. Application of Light Stimulation in Biomaterials for Peripheral Nerve Injury Repair

**Figure 3:**
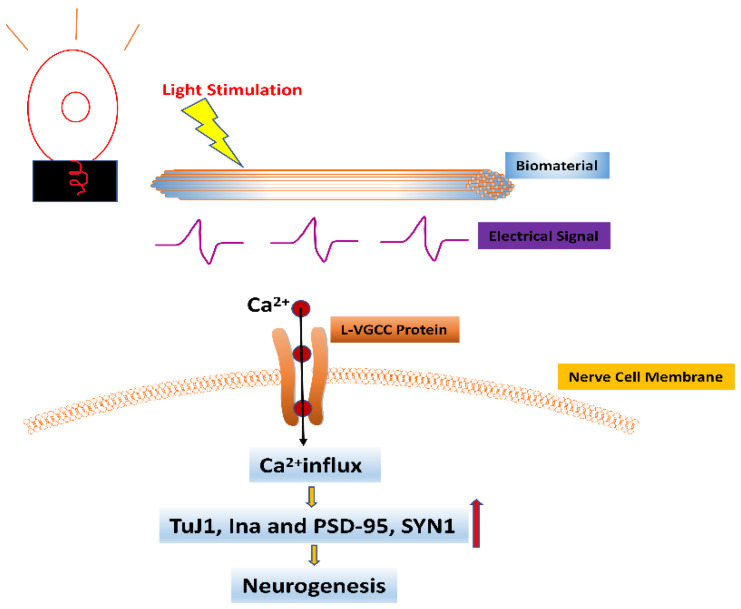
The effect of light stimulation combined with biomaterials on neurons. The electrical signal generated by the action of light stimulation on biomaterials promotes the opening of L-VGCC protein in nerve cell membranes, leading to Ca^2+^ inward flow, which in turn activates a series of intracellular cascades leading to the upregulation of expression levels of neural-associated genes β-Tubulin (TuJ1), neurofilament 66 (Ina), and neurogenic protein markers including postsynaptic density 95 (PSD-95) and synaptophysin 1 (SYN1), ultimately promoting neurogenesis. (Orange arrow represents promotion, red arrow represents increase).

Electrical signals stimulate the proliferation and differentiation of nerve cells and promote intercellular signaling [59,60]. Therefore, nerve scaffolds are often endowed with electroactive properties [61,62]. However, conventional conductive scaffolds often cause discomfort, inconvenience, or invasiveness [63]. Therefore, non-invasive alternative methods need to be developed. Photoelectric stimulators convert light into electrical signals, providing a wirelessly controlled and less invasive option for PNI repair [64]. The photodynamic scaffold has unique advantages such as being non-invasive and coming with high controllability [65,66]. Wu et al. established a photoconductive bio-interface [67]. The study found that under LED irradiation, axon growth and neuronal differentiation at biological interfaces are closely related to photoconductive stimulation, and the concentration of Ca^2+^ in PC12 cells was enriched [37]. In addition, some scholars integrated biophysical cues such as anisotropic topography guidance and photocatalyst g-C_3_N_4_ to form a three-dimensional polymer scaffold, which generates electricity and accelerates neuronal growth under visible light irradiation [12]. The study speculated that the mechanism of action on neuronal cells may be related to the activation of Ca^2+^ channels in the cell membrane [68] or may regulate neuronal synapse growth by triggering reactive oxygen species (ROS) production through the photocatalytic system of g-C_3_N_4_, similar to direct electric field stimulation [69]. This speculation provides ideas for the next step in exploring the mechanism of photo-conduction. To clarify the mechanism further, Sun et al., prepared electropolymerized pyrrole (PPy)/lanthanide-doped conversion nanoparticle (UCNP) photoelectrodes [70]. The study found that the rare earth elements released by UCNPs in the photoelectrode increased the level of ROS in PC12 cells through the extracellular signal-regulated kinase (ERK) signaling pathway, thereby realizing the growth-promoting effect of neurons [25]. It was also found that the mechanism by which the PCL-Col-P3HT scaffold promotes the directional growth of PC12 neurons under light stimulation involves an electrical signal being generated under light stimulation that promotes the opening of L-VGCC protein, leading to Ca^2+^ inward flow, which activates a series of intracellular cascade reactions and ultimately promotes neural regeneration of neuronal cells (as shown in Figure 3) [71]. The above two studies describe the mechanism of action of optoelectronic electrodes in stimulating nerve cell growth and development, but neither was verified in vivo, nor did they illustrate the lifetime of the electrodes. To solve these problems, Ejneby et al., developed a self-locking ultra-thin cuff that can precisely control stimuli by varying light intensity and pulse duration [72]. The cuff operated robustly for more than 100 days in an animal sciatic nerve model under laser irradiation without harmful histological changes, indicating that the device has good stability, biocompatibility, and high performance.

In conclusion, being able to regulate the light pulse intensity precisely makes it safer and more effective, and the long-term stable operation of the photodynamic scaffold is helpful for the treatment of significant gaps in PNI; however, there are still big problems with applying it in the clinic because, at present, it requires a light stimulator outside the body to generate a specific light source to achieve a therapeutic effect. In addition, only light in the 620 nm–800 nm region has good penetration depth in soft tissue such as skin, muscle, and adipose tissue [73]. The penetration of other wavelength ranges is limited. Therefore, how the visible light in daily life can be effectively and reasonably utilized is still a big topic, and it may be of great practical significance in the future by exploring effective visible light collection devices or designing devices that can convert visible light wavelengths to an appropriate scope and then apply them to the treatment of PNI repair.

## 5. Application of Magnetic Composite Biomaterials in Peripheral Nerve Injury Repair

Magnetic composite biomaterials have made significant progress in disease treatment [74]. Therefore, it is also desirable to explore their application in PNI repair. One study showed that low-frequency magnetic fields can change the direction of neuron growth, and the underlying mechanism may be related to mechanical forces acting on charged particles and macromolecules in and around neurons [75]. According to this principle, many studies explored the concept of using magnetic nanoparticles to make magnetic composite biomaterials to stimulate and/or guide neurite growth in the desired direction [76,77]. Glaser et al., prepared a hybrid scaffold of xanthan gum and magnetic nanoparticles (XCA/mag). This study found that the local magnetic field inherent in the magnetite particles on the scaffold promoted the expression of synaptophysin of embryonic stem cells and increased bioelectric transmission, which suggests that the magnetic complexion can provide a suitable microenvironment for neuronal growth [13]. In addition, some scholars mixed magnetic nanoparticles in collagen hydrogels, and neurons cultured on the mixed hydrogels formed elongated co-oriented morphologies influenced by the aggregation of magnetic particles into particle strings [78]. Another study showed that hydrogels containing magnetic alginate microparticles (MAMs) could guide and promote the growth of Schwann cells [79]. All the above studies confirmed the directional guidance of magnetic particles on nerve cells but unfortunately did not explore the mechanism of magnetism on nerve cells. To solve this problem, Tay et al., produced a 3D magnetic hydrogel composed of hyaluronic acid (H.A.), which can promote the growth of DRG neurites by inducing the inward flow of Ca^2+^ through the activation of mechanically sensitive endogenous PIEZO2 and TRPV4 ion channels [80]. In addition, nano-hydroxyapatite-coated magnetic nanoparticles can improve the vitality of nerve cells and axon elongation by activating the netrin-1 signaling pathway and directionally guiding the growth of DRG neurons [81]. It was also found that the reduced graphene oxide/collagen 3D nanocomposite hydrogel modified by magnetic nanoparticles can encapsulate neuroblastoma cells (SH-SY5Y), induce the directional growth of SH-SY5Y, and promote the expression of tyrosine hydroxylase, further promoting their maturation and differentiation [82]. To verify the effect of a magnetic composite material in vivo, researchers in one study produced a magnetically aligned regenerative tissue-engineered electronic nerve interface (MARTEENI) and implanted it within a rat sciatic-nerve transection model. It was found that the interface promoted the growth of nerve axons [83].

Magnetic composite biomaterials can accelerate the maturation and differentiation of neurons by guiding nerve cells to avoid uncontrolled growth and towards directional growth from the proximal end of the nerve stump to the distal end and by promoting and accelerating the repair of PNI. In addition, magnetic nanoparticles are easy to obtain, and at the same time, the preparation of magnetic composite biomaterials is simple compared to other neuro biomaterials; therefore, they are valuable for clinical PNI repair. However, considering the neurotoxicity and low magnetization characteristics of the existing magnetic nanoparticles [81], it is hoped that new, safe, highly magnetic, and biodegradable magnetic nanoparticles can be explored or synthesized in the future to increase the feasibility of clinical applications.

## 6. Application of Magnetic Fields in Biomaterials for Peripheral Nerve Injury Repair

**Figure 4:**
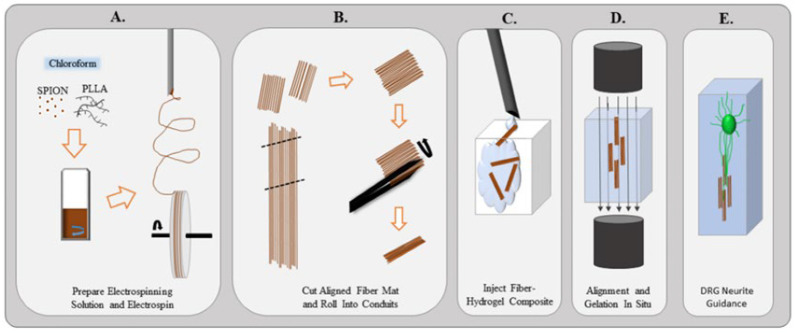
(**A**) Superparamagnetic iron oxide nanoparticles are mixed into the PLLA electrospinning solution and chloroform. Once dissolved, the solution is created an aligned fiber mat. (**B**) Mat is removed from the wheel and cut into segments. Using forceps, the fiber mats are rolled into a small conduit. (**C**) Small conduits and hydrogel are injected into a chamber. (**D**) Magnetic field is applied to orient the fibers within the hydrogel in situ. (**E**) Magnetic field is removed and the fibers remain aligned to guide neurites extending from the dorsal root ganglion. Adopted with permission from ref. [88].

The safety and effectiveness of magnetic fields (M.F.s) in regulating cell biological characteristics have been fully proven [27,84,85]; moreover, magnetic composite biomaterials also promote neuronal growth. It was found that different cell types have different sensitivities to M.F.s and that higher magnetic field strengths are likely to lead to significant apoptosis of neural cells and impair the viability of S.C.s [86]. Therefore, it is significant to study how to improve the safety and effectiveness of PNI repair through the magnetic modulation of magnetic nanocomposites by M.F.s. One group of scholars fabricated a super-paramagnetic responsive PLGA gelatin magnetite scaffold and found that the combination of the scaffold and magnetic field promotes the growth of BMSCs [87]. In addition, another study found that the synergistic effect of magnetic nanocomposites and a magnetic field could provide a regulatable regenerative microenvironment that supports the adhesion, diffusion, and proliferation of S.C.s [86].Furthermore, Johnson et al., fabricated a scaffold containing super-paramagnetic iron oxide nanoparticles (SPIONs) and found that the combination of the scaffold and an external magnetic field acted as a directional guide and facilitator of nerve axon growth (as shown in Figure 4) [88]. The study created a simple method to fabricate a magnetic fiber scaffold. However, the effect of appropriate M.F.s on neuronal growth has not been studied. To solve these problems, some scholars grafted SPION onto the surface of a PLLA electrospinning fiber to form fiber composites [89]. A static M.F. or alternating M.F. and linearly moving magnet were used to determine the optimal M.F.s’ stimulations to promote neuron growth. It was found that the grafting method in this study increased the action of SPION on neuronal cell membranes and contributed to axon extension, and the alternating magnetic field had the best effect on neurite growth [89]. To verify their effects in vivo further, the combined effect of a magnetic scaffold (M.G.) and M.F. on the activity of S.C.s and the repair of sciatic nerve injury in rats was explored [90]. The study showed that the combined effect could promote the connection of more axons from the proximal to the distal end of the nerve dissection via M.G. and re-innervate the target muscle, as well as regulate microvascular growth by promoting the survival of S.C.s and up-regulating vascularization-related genes, which ultimately promotes axonal regeneration and good recovery of nerve function.

The combined effect of M.F.s and magnetic biomaterials can more safely and reliably regulate the promoting effect of magnetism on neurons, thereby improving the therapeutic effect of PNI repair. Therefore, theoretically, it has clinical value. In addition, although studies found that alternating magnetic fields have the best effect on PNI, more research is needed to confirm the specific application parameters of the magnetic stimulation of neurons, such as mode of action, duration of action, frequency of use, etc. This combined effect should be applied to animal research, and a clearer and more definite mechanism of action should be explored to provide practical value for future clinical applications. Therefore, this combined effect is still a long way from clinical application.

## 7. Conclusions and Perspectives

This paper reviews the latest progress in the therapeutic effects and related mechanisms of PNI combined with various physical stimuli such as E.S., mechanical stimulation, light stimulation, and magnetic stimulation combined with biomaterials (as shown in Table 1). E.S. has received the most attention and progressed the fastest due to the potential advantages of E.S. for the treatment of PNI because the nerve fibers have the function of conducting electrochemical signals and are now developing in the direction of built-in miniaturization, flexibility, bio-absorbability, and long therapeutic durations. N.G. scaffolds can reasonably convert the mechanical energy of the movement of various body systems into electrical signals for the treatment of PNI, with obvious convenience. Among them, the use of autonomic nerves to control breathing movement to achieve more accurate regulation of the magnitude and rhythm of electrical signals suitable for peripheral nerves is the latest research direction, which has reference significance for the future treatment of PNI. Light stimulation is less invasive in the treatment of PNI and has the advantage of being adjustable. However, it requires the assistance of a photoelectric converter to convert light energy into electrical signals before it can function. Therefore, there is still room for improvement in terms of the convenience of clinical application. The combined effect of magnetic stimulation and biomaterials is a recently explored topic. The safety and optimal parameters of magnetic field stimulation for the in vivo use of magnetic materials still needs to be investigated for convenience. Previous studies found that thermally responsive biomaterials can promote the growth of S.C.s and axons in vitro, showing potential for application in PNI repair [91,92,93,94]. It is necessary to combine them further in vivo to verify their mechanisms and effects in the future. The combined treatment of physical stimulation and biomaterials significantly improved the repair effect after PNI, and even achieved similar effects to autologous transplantation in some ways. Coupled with the convenience and safety of physical stimulation (when compared with the addition of biomaterials), it may be considered that physical stimulation combined with biomaterials will be the general trend of PNI repair in the future.

Although the combination of the above physical stimulation factors and neural repair biomaterials has good application prospects, there are still some problems to be worked on, including (1) the optimization of the operational life of self-powered devices and the development of biodegradable battery or electrode materials; (2) physical stimulation combined with nerve conduits containing various biological and physical cues, such as biomimetic intraluminal fillers, conductive coating pathways, and neurotrophic factor transport; (3) the combined use of various physical stimuli to explore the best combination for PNI repair; and (4) the use of physical stimulation combined with biomaterials for significant gap nerve defects (≥3 cm) in animal models to prepare for human applications. Overall, physical stimulation is an effective treatment for PNI, and more consideration needs to be given to combining it with multiple stimulation factors that promote PNI repair in the future to achieve better results. 

## Figures and Tables

**Table 1:** Advantages and disadvantages of different methods of physical stimulation combined with biomaterials.

Authors	Physical Stimulation	Methods	Advantages	Disadvantages
Wang et al. [35]	Electrical stimulation	in vitro/vivo	①Self-powered ②Biodegradable ③No external equipment	①Short lifespan
Sun et al. [32]	Electrical stimulation	in vitro/vivo	①Self-powered ②Biodegradable ③No external equipment	①Foreign body reaction
Mohseni et al. [10]	Electrical stimulation	in vitro	①Self-powered ②No external equipment	①Unknown
MacEwan et al. [40]	Electrical stimulation	in vivo	①Functional assessment	
			②Wirelessly controlled ③Self-powered	①Unknown
McAvoy et al. [46]	Electrical stimulation	in vivo	①Stretchable ②Epimysial recording ἱWirelessly controlled ④Self-powered	①Nonbiodegradable
Guo et al. [47]	Electrical stimulation	in vitro/vivo	①Wirelessly controlled ②Biodegradable ③Self-powered	①Short lifespan ②Requires external equipment
Choi et al. [33]	Electrical stimulation	in vitro/vivo	①Stretchable ②Wirelessly controlled ③Biodegradable ④Long lifespan ⑤Self-powered	①Requires external equipment
Cuttaz et al. [45]	Electrical stimulation	in vitro	①Stretchable ②Self-powered	①Unknown
Koo et al. [50]	Electrical stimulation	in vivo	①Wirelessly controlled ②Biodegradable ③Self-powered	①Requires external equipment
Charthad et al. [43]	Electrical stimulation	in vitro	①Wirelessly controlled ②High current intensity ③Self-powered	①Requires external equipment ②Nonbiodegradable
Han et al. [41]	Electrical stimulation	in vitro	①Wirelessly controlled ②Self-powered	①Requires external equipment
Hernandez-Reynoso et al. [42]	Electrical stimulation	in vitro	①Wirelessly controlled ②Self-powered	①Requires external equipment ②Nonbiodegradable
Qian et al. [56]	Mechanical energy	in vivo	①No external equipment ②Self-powered ③Long lifespan	①Unknown
Jin et al. [58]	Mechanical energy	in vivo	①Self-regulated E.S. ②Self-powered ③Long lifespan	①Unknown
Guo et al. [54]	Mechanical energy	in vitro/vivo	①Self-powered ②Biodegradable	①Unknown
Ejneby et al. [72]	Light stimulation	in vivo	①Long lifespan ②Stretchable ③Wirelessly controlled ④Self-powered	①Requires external equipment
Wu et al. [64]	Light stimulation	in vitro	①Wirelessly controlled ②Self-powered	①Unknown
Sun et al. [95]	Light stimulation	in vitro	①Wirelessly controlled ②Self-powered	①Requires external equipment
Zhang et al. [12]	Light stimulation	in vitro	①Wirelessly controlled ②Self-powered	①Unknown
Tang et al. [71]	Light stimulation	in vitro	①Wirelessly controlled ②Self-powered	①Requires external equipment
Tay et al. [80]	Magnetic composite	in vitro	①Magnetomechanical neuromodulation	①Unknown
Liu et al. [81]	Magnetic composite	in vitro	①Directional guidance	①Unknown
Zuidema et al. [76]	Magnetic composite	in vitro	①Directional guidance	①Unknown
Santhosh et al. [82]	Magnetic composite	in vitro	①Directional guidance ②Promoting neuron differentiation	①Unknown
Glaser et al. [13]	Magnetic composite	in vitro	①Facilitates synapse formation	①Unknown
Antman-Passig et al. [78]	Magnetic composite	in vitro	①Directional guidance	①Unknown
Singh et al. [79]	Magnetic composite	in vitro	①Directional guidance ②Promoting neuron differentiation	①Unknown
Kasper et al. [83]	Magnetic composite	in vitro/vivo	①Directional guidance	①Nonbiodegradable
Funnell et al. [89]	Magnetic fields	in vitro	①Wirelessly controlled	①Requires external equipment
Johnson et al. [88]	Magnetic fields	in vitro	①Wirelessly controlled	①Requires external equipment
Liu et al. [90]	Magnetic fields	in vitro/vivo	①Wirelessly controlled ②Directional guidance	①Requires external equipment
Liu et al. [86]	Magnetic fields	in vitro	①Wirelessly controlled	①Requires external equipment
Ghorbani et al. [87]	Magnetic fields	in vitro	①Wirelessly controlled	①Requires external equipment

## Data Availability

Not applicable.
